# Cytotoxicity of Biologically Synthesized Silver Nanoparticles in MDA-MB-231 Human Breast Cancer Cells

**DOI:** 10.1155/2013/535796

**Published:** 2013-07-08

**Authors:** Sangiliyandi Gurunathan, Jae Woong Han, Vasuki Eppakayala, Muniyandi Jeyaraj, Jin-Hoi Kim

**Affiliations:** ^1^Department of Animal Biotechnology, Konkuk University, 1 Hwayang-dong, Gwangjin-gu, Seoul 143-701, Republic of Korea; ^2^GS Centre for Life Sciences, Sundarapuram, Coimbatore, Tamil Nadu 641024, India

## Abstract

Silver nanoparticles (AgNPs) have been used as an antimicrobial and disinfectant agents. However, there is limited information about antitumor potential. Therefore, this study focused on determining cytotoxic effects of AgNPs on MDA-MB-231 breast cancer cells and its mechanism of cell death. Herein, we developed a green method for synthesis of AgNPs using culture supernatant of *Bacillus funiculus*, and synthesized AgNPs were characterized by various analytical techniques such as UV-visible spectrophotometer, particle size analyzer, and transmission electron microscopy (TEM). The toxicity was evaluated using cell viability, metabolic activity, and oxidative stress. MDA-MB-231 breast cancer cells were treated with various concentrations of AgNPs (5 to 25 **μ**g/mL) for 24 h. We found that AgNPs inhibited the growth in a dose-dependent manner using MTT assay. AgNPs showed dose-dependent cytotoxicity against MDA-MB-231 cells through activation of the lactate dehydrogenase (LDH), caspase-3, reactive oxygen species (ROS) generation, eventually leading to induction of apoptosis which was further confirmed through resulting nuclear fragmentation. The present results showed that AgNPs might be a potential alternative agent for human breast cancer therapy.

## 1. Introduction

Breast cancer is the second most common cause of cancer death in women [1, 2]. Many cancers initially respond to chemotherapy, and later they develop resistance [3–5]. Currently available chemopreventives and chemotherapeutic agents cause undesirable side effects [6, 7]; therefore developing a biocompatible and cost effective method of treatment for cancer is indispensable. The development of nanotechnology has been a boon to mankind as its significance paved the way for several applications in therapeutics [8], catalysis [9], microelectronics, biosensing devices [10], air and water purifiers, paints [11], and so forth. Recently, AgNPs have gained much interest among the emerging nanoproducts in the field of nanomedicine due to their unique properties and obvious therapeutic potential in treating a variety of diseases, including retinal neovascularization [12–15]. The nanoparticles can be synthesized by physical, chemical, and biological methods. The physical methods are initially used to give a low yield [16]. Chemical methods use various chemical agents to reduce metallic ions to nanoparticles. This comprises certain drawbacks as there will be use of toxic chemicals and generation of hazardous byproducts [16]. In the medical aspects, applications of nanoparticles increased tremendously only when the biological approach for nanoparticle synthesis came into focus. The various resources available naturally for green synthesis of nanoparticles are plants, plant products, bacteria, fungi, algae, yeast, and viruses [17]. Though there is a large platform for the green synthesis of nanoparticles, the most commonly preferred way is the bacterial synthesis, as they are easy to handle, and genetic manipulation is also possible [13, 18, 19]. 

The major implication of this biological approach is simple and less time consuming. In addition to this the high yield, low toxicity, low cost, and its biocompatibility add to its value [20]. An additional advantage is that the size of the nanoparticles synthesized can also be easily controlled by various controlling parameters like pH, temperature [13], and the use of stabilizers to prevent aggregation is not required as the proteins in the system act as stabilizers [14]. Nanoparticles with smaller radius of curvature have higher catalytic activity; hence angular shapes are preferable due to their smaller radii of curvature compared to spherical particles of the same volume. Several research groups have successfully demonstrated the superior antimicrobial efficacy of AgNPs either as they are or in composites with polymer [21–25]. In addition, our group and another research group demonstrated that AgNPs have potential cytotoxicity against cancer [15, 26, 27] and antiangiogenic property in microvascular endothelial cells [28, 29].

Recently, Rani et al. [27] reported that AgNPs inhibit proliferation of human glioblastoma cells. Franco-Molina et al. [30] evaluated the effects of colloidal silver on MCF7 human breast cancer cells. Sanpui et al. [31] demonstrated that AgNPs, not only disrupting normal cellular function and but also affecting the membrane integrity, induced various apoptotic signaling genes of mammalian cells leading to programmed cell death. Hsin et al. [32] reported that AgNPs induced apoptosis in NIH3T3 cells by heightening the ROS generation and activated JNK pathway leading to mitochondria-dependent apoptosis. Recent studies have shown that AgNPs accumulation in the liver could induce cytotoxicity via oxidative cell damage [32–34]. Reactive oxygen species (ROS) are continually generated and eliminated in biological systems. They play an important role in a variety of normal biochemical functions, and abnormality in their function results in pathological processes. Excessive production of ROS in the cell is known to induce apoptosis [35, 36]. ROS generation has been shown to play an important role in apoptosis induced by treatment with AgNPs [27, 37, 38]. 

A number of studies have reported that AgNPs may induce cytotoxicity in phagocytosing cells, such as not only mouse peritoneal macrophages but also human monocytes [38–40]. Further studies suggested that the cytotoxic effects were induced by reactive oxygen species (ROS) resulting in cellular apoptosis, at least low concentrations and short incubation times [37, 41–43]. The production of ROS has also been implicated in DNA damage caused by AgNPs, which was reported in a number of in vitro studies [27, 38, 44]. Caspase-3 is one of the key molecules in apoptosis, and its activation is often considered as the point of no return in apoptosis [45]. Activation of caspase-3 results in the cleavage of (inhibitor of caspase-activated DNAse) ICAD and translocation of (caspase activated DNAse) CAD to the nucleus, ultimately resulting in DNA fragmentation. The most prominent event in the early stages of apoptosis is internucleosomal DNA cleavage by endonuclease activities [46]. Previous studies suggested that AgNPs treated cancer cell, and noncancer cells revealed enhanced caspase-3 activity and formation of significant DNA laddering [14, 15, 47]. 

Currently, a variety of cytotoxic agents have been used in the treatment of breast cancer, such as doxorubicin, cisplatin, and bleomycin [30, 48]. Although usage of doxorubicin, cisplatin, and bleomycin provides beneficial effect, but the efficacy and demerits are uncertain [30]. Therefore, it is necessary to find novel therapeutic agents against cancer, which are biocompatible and cost effective. Therefore, this study was designed to synthesize AgNPs using biological system and to evaluate potential toxicity and the general mechanism of biologically synthesized AgNPs in MDA-MB-231 human breast cancer cells.

## 2. Materials and Methods

### 2.1. Materials

Penicillin-streptomycin solution, trypsin-EDTA solution, RPMI-1640 medium, Dulbecco's modified Eagle's medium (DMEM/F-12), and 1% antibiotic-antimycotic solution were obtained from Life Technologies GIBCO, Grand Island, NY, USA. Fetal bovine serum (FBS), in vitro toxicology assay kit, was purchased from Sigma-Aldrich (St. Louis, MO, USA).

### 2.2. Synthesis of AgNPs

Luria-Bertani broths were prepared and used as described earlier [13]. *B. funiculus* cultures were obtained from the GS Center for Life Sciences, Coimbatore, India. The novel bacteria were isolated from industrial wastewater, and sequence has been submitted at GenBank. The strain was grown aerobically at 37°C in LB medium. Synthesis of AgNPs was carried out according to the method described previously [13]. Briefly, bacteria were grown in a 500 mL Erlenmeyer flask that contained LB broth without NaCl or nitrate medium. The flasks were incubated for 21 h in a shaker set at 120 rpm and 37°C. After the incubation period, the culture was centrifuged at 10,000 rpm/min and the supernatant used for the synthesis of AgNPs. Three vials the first containing AgNO_3_ (Sigma, USA, 99.9% pure) without the supernatant, the second containing only the culture supernatant, and the third containing the supernatant and AgNO_3_ solution at a concentration of 1 mM were incubated for 60 min at 40°C. The extracellular synthesis of AgNPs was monitored by visual inspection of the test tubes for a change in the color of the culture medium from a clear, light yellow to brown, and by measurement of the peak exhibited by AgNPs in the UV-vis spectra the synthesis of nanoparticles was confirmed. 

### 2.3. Cell Culture

MDA-MB-231 human breast cancer cells were kindly provided by Professor Ssang-Goo, Department of Animal Biotechnology, Konkuk University, and were maintained in Dulbecco's modified Eagle's medium (DMEM) supplemented with 10% fetal bovine serum (FBS) and 1% antibiotic-antimycotic solution. Cells were grown to confluence at 37°C and 5% CO_2_ atmosphere. All experiments were performed in 6-well plates, unless stated otherwise. Cells were seeded onto the plates at a density of 1 × 10^6^ cells per well and incubated for 24 h prior to the experiments. The cells were washed with (phosphate buffered saline, pH 7.4) PBS and incubated in fresh medium containing different concentrations of AgNPs dissolved in water. 

### 2.4. Characterization of AgNPs

Characterization of synthesized AgNPs particles was carried out according to the methods described previously [13, 49]. The AgNPs were primarily characterized by UV-visible spectroscopy. Ultraviolet-visible (UV-vis) spectra were obtained using a WPA (Biowave II), Biochrom, Cambridge, UK. The particle size of dispersions was measured by Zetasizer Nano ZS90 (Malvern Instruments, Ltd., UK). Transmission electron microscopy (TEM, JEM-1200EX) was used to determine the size and morphology of AgNP. 

### 2.5. Cell Viability Assay

The cell viability assay was measured using the 3-(4,5-dimethylthiazol-2-yl)-2,5-diphenyltetrazolium bromide dye reduction assay which was performed to determine the cytotoxic effect of the AgNPs at various concentrations. Briefly, the MDA-MB-231 cells were plated onto 96-well flat bottom culture plates with various concentrations of AgNPs. All cultures were incubated for 24 h at 37°C in a humidified incubator. After 24 h of incubation (37°C, 5% CO_2_ in a humid atmosphere), 10 *μ*L of MTT (5 mg/mL in PBS) was added to each well, and the plate was incubated for a further 4 h at 37°C. The resulting formazan was dissolved in 100 *μ*L of DMSO with gentle shaking at 37°C, and absorbance was measured at 595 nm with an ELISA reader (Spectra MAX; Molecular Devices, USA). The results were given as the mean of three independent experiments. Concentrations of AgNPs showing a 50% reduction in cell viability (i.e., IC50 values) were then calculated. 

### 2.6. Membrane Integrity

Cell membrane integrity of MDA-MB-231 cells was evaluated by determining the activity of lactate dehydrogenase (LDH) leaking out of the cell according to the manufacturer's instructions (in vitro toxicology assay kit, TOX7, Sigma, USA). The LDH assay is based on the release of the cytosolic enzyme, LDH, from cells with damaged cellular membranes. Thus, in cell culture, the course of AgNPs induced cytotoxicity was followed quantitatively by measuring the activity of LDH in the supernatant. Briefly, cells were exposed to various concentrations of AgNPs for 24 h, then 100 *μ*L per well of each cell-free supernatant was transferred in triplicates into wells in a 96-well, and 100 *μ*L of LDH assay reaction mixture was added to each well. After 3-hour incubation under standard conditions, the optical density of the color generated was determined at a wavelength of 490 nm using a Microplate Reader. 

### 2.7. Determination of ROS

Intracellular reactive oxygen species (ROS) were measured based on the intracellular peroxide-dependent oxidation of 2′,7′-dichlorodihydrofluorescein diacetate (DCFH-DA, Molecular Probes, USA) to form the fluorescent compound 2′,7′-dichlorofluorescein (DCF), as previously described [50]. Cells were seeded onto 24-well plates at a density of 5 × 10^4^ cells per well and cultured for 24 h. After washing twice with PBS, fresh medium containing 8.7 *μ*g AgNPs or 1 mM H_2_O_2_ was added, and the cells were incubated for 24 h. For control, the cells were added 20 *μ*M of DCFH-DA, and incubation continued for 30 min at 37°C. The cells were rinsed with PBS, 2 mL of PBS was added to each well, and fluorescence intensity was determined with spectrofluorometer (Gemini EM) with excitation at 485 and emission at 530 nm. For control, the cells grown in 24-well plates for 24 h were added an antioxidant N-acetyl-L-cystein (NAC, 5 mM) for 1 h prior to exposing them to 17.4 to 8.7 *μ*g/mL AgNPs or 1 mM H_2_O_2_ for 12 h. 20 *μ*M of DCFH-DA was then added, and the cells were incubated for 30 min at 37° before measuring changes of DCF fluorescence as described. 

### 2.8. Measurement of Caspase-3 Activity

The cells were treated with 8.7 *μ*g AgNPs or purified caspase-3 or inhibitor for 24 h. The activity of caspase-3 was measured in MDA-MB-231 cells using a kit from Sigma (St. Louis, MO, USA) according to the manufacturer's instructions. Cells were washed with ice-cold PBS and lysed with 100 *μ*L of lysis buffer [50 mM Tris-HCl (pH 7.5), 150 mM NaCl, 1 mM EGTA, 1 mM NaF, 1% Nonidet P-40, 1 mM PMSF, and protease inhibitor cocktail] for 30 min at 4°C. Protein extracts were collected after centrifugation at 14,000 rpm for 10 min. Protein concentration was determined using the Bio-Rad protein assay kit (Bio-Rad, USA). Equal amounts (50 *μ*g) of protein extracts were mixed with assay buffer [20 mM HEPES (pH 7.4), 100 mM NaCl, 0.1% CHAPS, 10 mM DTT, 1 mM EDTA, and 10% sucrose], added to 96-well Microtiter plates, and incubated with the caspase-3 substrate (acetyl-Asp-Glu-Val-Asp p-nitroanilide (Ac-DEVD-pNA) and caspase-3 inhibitor (Ac-DEVD-CHO) for 1 h and the absorbance read at 405 nm. The colorimetric assay is based on the hydrolysis of caspase-3 substrate by caspase-3, resulting in the release of the p-nitroaniline (pNA) moiety. The concentration of the pNA released from the substrate is calculated from the absorbance values at 405 nm. The assay was done with noninduced cells and also in the presence of purified caspase-3 and caspase-3 inhibitor (Ac-DEVD-CHO) for a comparative analysis.

### 2.9. DNA Fragmentation Assay

MDA-MB-231 (10^6^ cells mL) were seeded in 6-well Microplates and treated with 8.7 *μ*g/mL of AgNPs. After 24 h of treatment, the culture medium was removed, and the cells were harvested by scraping with 1 mL of PBS and lysed with 500 *μ*L of lysis buffer [20 mM Tris-HCl (pH 8.0), 5 mM EDTA, 400 mM NaCl, 1% SDS, and 10 mg/mL proteinase K] for 1 h at 55°C. Fragmented DNA was extracted with phenol/chloroform/isoamyl alcohol (25 : 24 : 1 v/v/v), precipitated with ethanol, and resuspended in Tris-EDTA buffer (TE, pH 8.0) containing 20 *μ*g/mL RNase A. For quantitative analyses of DNA content, an equal amount of DNA was loaded and run on a 1.0% agarose gel containing 1 *μ*g/mL ethidium bromide at 70 V, and the DNA fragments were visualized by exposing the gel to ultraviolet light, followed by photography. For control, cells grown in 24-well plates for 24 h were added an antioxidant N-acetyl-L-cystein (NAC, 5 mM) for 1 h prior to exposing them to 8.7 *μ*M AgNPs. 

## 3. Results and Discussion

### 3.1. Extracellular Synthesis of AgNPs

**Figure 1:**
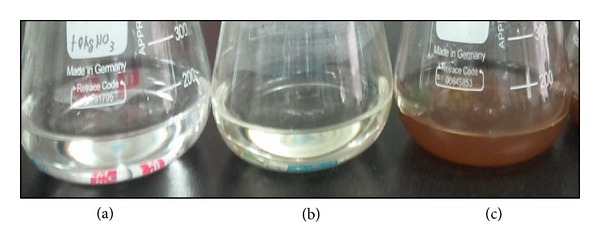
Synthesis of AgNPs by culture supernatant of *B. funiculus.* The figure shows flask containing samples of AgNO_3_ after exposure to 60 min (a), AgNO_3_ with the extracellular culture supernatant of *B. funiculus* (b), and AgNO_3_ plus supernatant of *B. funiculus* (c). It is observed that the color of the solution turned from colorless to brown after 1 h of the reaction, indicating the formation of AgNPs.

There are several physical and chemical methods that have been used for the synthesis of metallic nanoparticles [51]. The development of biological method is essential because it provides cost effective, environmentally friendly, and easy process for synthesis and application of metallic nanoparticles. Several microorganisms have the potential to interact with metal ions reducing them into metallic nanoparticles [52]. In this work, we took the advantage of using microorganism for synthesis of AgNPs using the culture supernatants of* B. funiculus*. As shown in Figure 1, the supernatants of *B. funiculus* were incubated with silver nitrate (1 mM). The appearance of a yellowish-brown color in the reaction vessels suggested the formation of colloidal AgNPs. Thus, it was evident that the metabolites excreted by the culture exposed to silver could reduce silver ions, clearly indicating that the reduction of the ions occurs extracellularly through reducing agents released into the solution by *B. funiculus.* The color change from yellow to brown provides a piece of evidence to support the synthesis of AgNPs, and also it is due to the excitation of surface plasmon vibrations, typical of AgNPs [13, 53–56].

**Figure 2:**
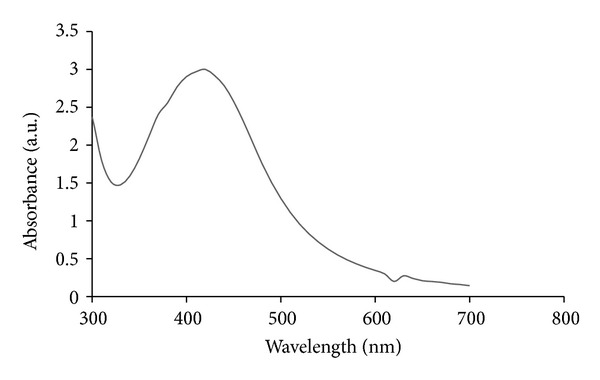
The absorption spectrum of AgNPs synthesized by *B. funiculus* culture supernatant. The absorption spectrum of AgNPs exhibited a strong broad peak at 420 nm, and observation of such a band is assigned to surface plasmon resonance of the particles. The samples were collected and were incubated with 1 mM silver nitrate solution. After the incubation period, the samples were visualized in UV-vis spectra.

Further, the AgNPs were characterized by UV-visible spectroscopy. The UV-visible absorption spectra of the cell filtrates were measured in the range of 300–700 nm using a UV-visible spectrophotometer. This technique has been proved to be a very valuable and useful technique for the characterization of nanoparticles [53–55]. By using UV-visible spectroscopy, we could measure the diameter by the spectral response of silver nanoparticles; as the diameter increases, the peak plasmon resonance shifts to longer wavelengths and broadens. Additionally, UV-visible spectroscopy provides a mechanism to monitor how the nanoparticles change over time. A strong and broad surface plasmon peak located at 420 nm was observed for the AgNPs prepared using supernatants of* B. funiculus* (Figure 2). The band around 420 nm suggests that the particles were well dispersed without aggregation. Observation of the strong but broad surface plasmon peak has been well known in the case of various metal nanoparticles over a wide size range of 2–100 nm [53, 54].

### 3.2. Size Distribution Analysis by Dynamic Light Scattering (DLS)

**Figure 3:**
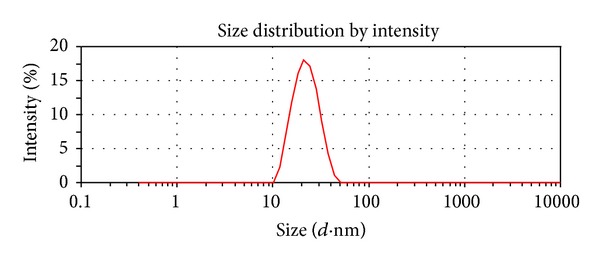
Size distribution analysis by DLS. The particle size distribution revealed that the particles range from 10–20 nm. The average particle size was found to be 20 nm.

To know the size of synthesized AgNPs, size distribution analysis was performed using dynamic light scattering in aqueous solution. It was found that the average size of AgNPs was 20 nm. Figure 3 shows that the particles range in size from 10 to 20 nm and possess an average size of 20 nm synthesized by *B. funiculus. *DLS results showed an average diameter of 20 nm and a low polydispersity index of less than 0.08 indicating that a monodisperse distribution of mostly a single and uniform size of species is present in solution.

Regarding the size of the AgNPs, several studies have been reported using a biological system such as using culture supernatant of *K. pneumonia* produces the size of particles range from 28.2 to 122 nm and possess an average size of 52.5 nm [55]. Similarly, both culture supernatant and biomass of *B. licheniformis *producesabout the size of 50 nm [49, 56]. Gurunathan et al. [13] reported that the particles range in size from 42.2 to 89.6 nm and possess an average size of 50 nm size of AgNP synthesized from culture supernatant of *E. coli* was 50 nm; using DLS and TEM. Shanshoury et al. [57] reported the extracellular biosynthesis of metallic AgNPs by the reduction of aqueous Ag^+^ using *Escherichia coli *ATCC 8739, *Bacillus subtilis *ATCC 6633, and *Streptococcus thermophilus *ESh1 and revealed the size range between 5 and 25 nm. 

### 3.3. Size and Morphology Analysis of AgNPs by Transmission Electron Microscopy (TEM)

**Figure 4:**
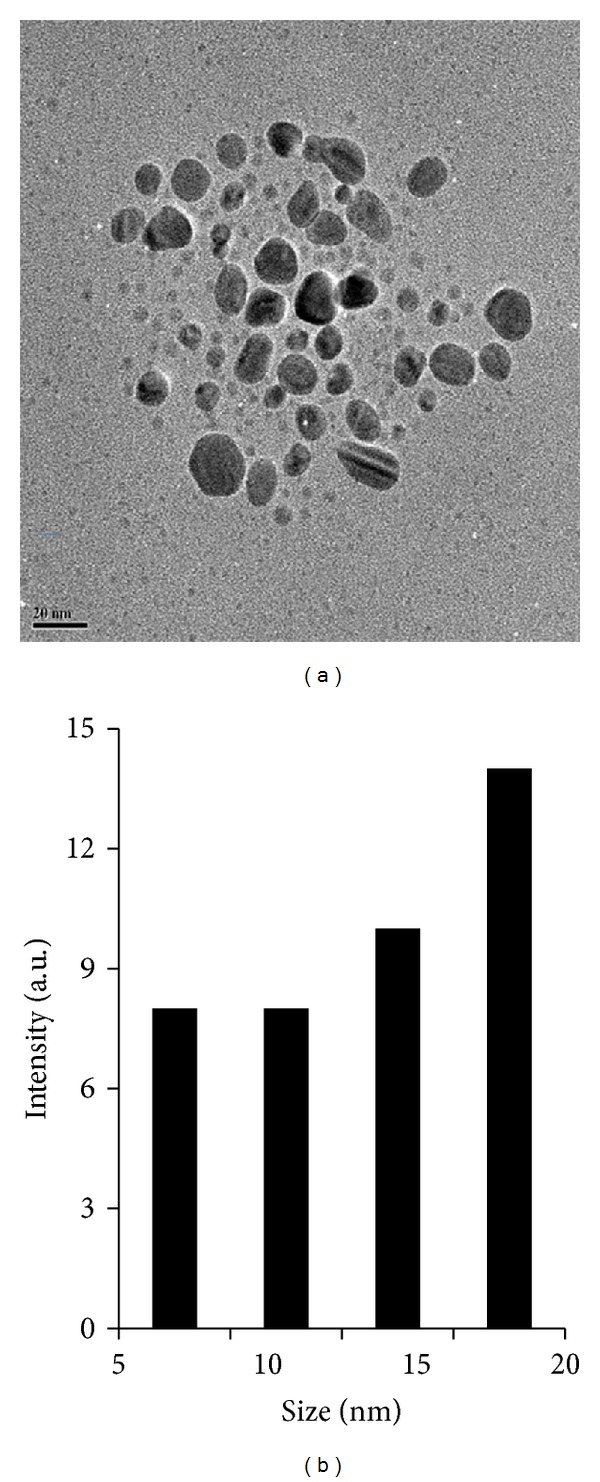
Size and morphology of AgNPs analysis by TEM. (a) Several fields were photographed and were used to determine the diameter of nanoparticles. The average range of observed diameter was 20 nm. (b) Particle size distributions from TEM image.

Further characterization of AgNPs was examined using TEM to know the size and morphology of AgNPs; the representative TEM image was shown in Figure 4 and indicates well-dispersed particles which are more or less spherical. The TEM analysis revealed that the particle size of silver particles shows that the particle size ranges from 10 to 20 nm. 

To determine their size distribution, we measured approximately more than 200 particles from various samples and represented them as the size distribution analysis (Figure 4(b)). The average size of the particles synthesized by *B. funiculus *is approximately 20 nm. A significant proportion of largely spherical AgNPs within the range of 20 nm were observed in TEM micrographs. The spherical particles are reasonably uniform and range in size from 10 to 20 nm and are in agreement with DLS data.

### 3.4. Dose-Dependent Cytotoxicity Effect of AgNPs in MDA-MB-231 Cells

**Figure 5:**
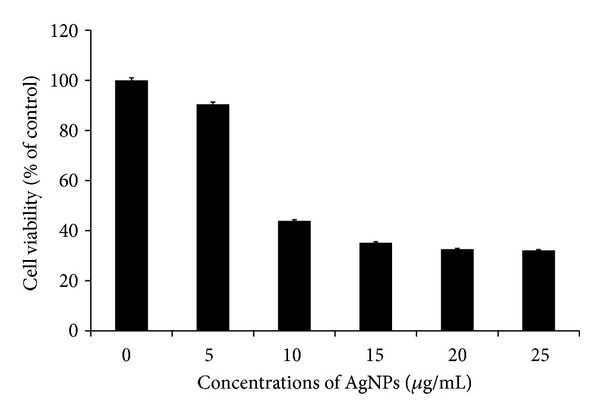
Effect of AgNPs on Cell viability of MDA-MB-231 cells. Cells were treated with AgNPs at various concentrations for 24 h, and cytotoxicity was determined by the MTT method. The results represent the means of three separate experiments, and error bars represent the standard error of the mean. Treated groups showed statistically significant differences from the control group by the Student's *t*-test (*P* < 0.05).

The cell viability assay is one of the important methods for toxicology analysis which explain the cellular response to a toxic materials, and it can provide information on cell death, survival, and metabolic activities [27]. Recently, Piao et al. [43] reported that AgNPs and AgNO_3_ showed cytotoxicity in a dose-dependent manner in human Chang liver cells; among these materials AgNPs showed higher cytotoxicity compared to AgNO_3_. AgNPs treated cells showed the decreased metabolic activity, which depends on nature of cell types and size of nanoparticles [58]. Franco-Molina et al. [30] reported that colloidal silver induced dose-dependent cytotoxic effect on MDA-MB-231 breast cancer cells. In our experiment, the cells were treated with various concentrations (0–25 *μ*g/mL) of AgNPs for 24 h, and the results suggest that AgNPs were able to reduce the cell viability of MDA-MB-231 cells in a dose dependent manner. At 24 h of treatment, AgNPs was found to be cytotoxic to the cells at concentrations of 10 *μ*g/mL and higher (Figure 5). In agreement with our results, other research groups have reported that cell viability was significantly reduced as a function of both culture time and AgNP concentration in human IMR-90 and U251 cells, mouse embryonic stem cells, and A549 lung cells [27, 44]. Our results suggest that the lowest concentration of AgNPs significantly inhibits the growth of cells. 

### 3.5. Impact of AgNPs on Membrane Integrity

**Figure 6:**
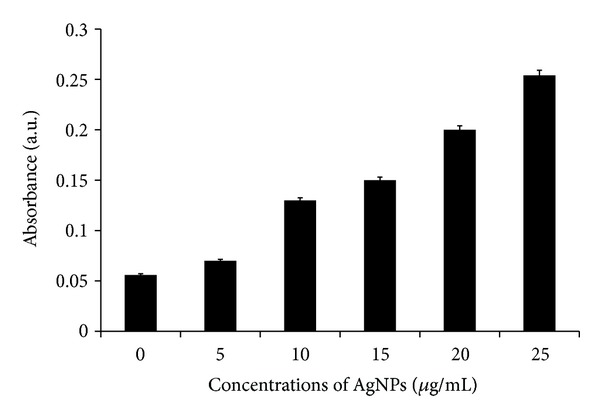
Effect of AgNPs on LDH activity in MDA-MB-231. LDH activity was measured by changes in optical densities due to NAD^+^ reduction which were monitored at 490 nm, as described in Materials and Methods Section, using the cytotoxicity detection lactate dehydrogenase kit. The results represent the means of three separate experiments, and error bars represent the standard error of the mean. Treated groups showed statistically significant differences from the control group by the Student's *t*-test (*P* < 0.05).

LDH is a soluble cytosolic enzyme, which is released into the extracellular medium because membrane damage consequently leads to apoptosis. It is widely accepted as an indicator of lytic cell death. The results show that cell membrane integrity in MDA-MB-231 cells was compromised in a dose dependent manner by AgNPs of 20 nm diameter (Figure 6). The inverse relationship between the LDH and the MTT cell viability results adds support to the accuracy of the data. In the LDH assay, as the concentration of the AgNPs increased, cells became progressively more cytotoxic, leading to a higher absorbance reading in the LDH assay and a decrease in absorbance in the MTT assay with a concurrent decrease in the percentage of viable cells. Park et al. [58] observed with various sizes of AgNPs that cell membrane integrity in L929 fibroblasts was compromised by all three AgNPs, with 20 nm AgNPs being more potent than 80 and 113 nm AgNPs. However the cell membrane integrity was affected slightly in RAW 264.7 macrophages. Song et al. [59] reported that water-soluble mPEG-SH-coated AgNPs decreased cell viability in dose- and time-dependent manners at dosage levels between 6.25 and 100.00 *μ*g/mL, caused membrane damage (LDH leakage), and decreased the activities of superoxide dismutase and glutathione peroxides. AgNPs induced the release of LDH in a concentration- and time-dependent manner, indicating that AgNPs reduced the membrane potential in A549 cells. Lee et al. [60] observed that the LDH level was increased 210% when cells were cultivated for 48 h in the culture medium containing AgNPs at 100 *μ*g/mL. Hussain et al. [33] demonstrated that exposure to AgNPs for 24 h resulted in a concentration-dependent increase in LDH leakage and exhibited a significant cytotoxicity at 10–50 *μ*g/mL in BRL 3A rat liver cells.

### 3.6. Determination of IC50 Values of AgNPs

To focus on the cytotoxic effect of particular concentration, the half maximal inhibitory concentration (IC50) was calculated as the concentration required to inhibit the growth of tumor cells in culture by 50% compared to the untreated cells. AgNPs at 8.7 *μ*g/mL decreased the viability of MDA-MB-231 cells to 50%, and this was chosen as the IC50. Longer exposures resulted in additional toxicity to the cells. These results demonstrate that AgNPs mediate a concentration-dependent increase in toxicity. Because 8.7 *μ*g/mL concentrations of AgNPs were found to be the IC50, further experiments were carried out using this concentration, to show the effect of AgNPs against MDA-MB-231 cells. Gopinath et al. [26] investigated the molecular mechanism of 10–15 nm size of AgNP mediated cytotoxicity in BHK21 (noncancer) and HT29 (cancer) cells, and they observed that 27 *μ*g/mL seems to be IC50. Zanette et al. [61] investigated the effects of AgNPs on skin using the human-derived keratinocyte HaCaT cell line model and suggested that AgNPs caused a concentration- and time-dependent decrease of cell viability, with IC50 values of 6.8 ± 1. *μ*M (MTT assay) and 12 ± 1.2 *μ*M (SRB assay) after 7 days of contact. The IC50 results obtained from our studies are comparable with earlier reports, and synthesized AgNPs show more efficacy than earlier reports. However, the action of AgNP depends on size, shape, conditions of media, and type of cells are and also dose and time dependent.

### 3.7. Effect of AgNPs in Cellular Reactive Oxygen Species

Oxidative stress is one of the key mechanisms of toxicity related to nanoparticle exposure [62]. The interaction between AgNPs and mammalian cells can induce oxidative stress by inducing the cellular ROS production so that it exceeds the cellular antioxidant capacity [27]. Oxidative stress plays important roles in a variety of normal biochemical functions, and abnormality in their function results in pathological processes. Excessive production of ROS in the cell is known to induce apoptosis [63, 64]. ROS generation has been shown to play an important role in apoptosis induced by treatment with AgNPs [27, 37, 38]. Our studies provided evidence for a molecular mechanism of AgNPs inducing generation of ROS, and it could be one of the factors for apoptosis. Earlier studies show that AgNPs could induce generation of ROS in macrophages [58] and human Chang liver cells [43]. 

**Figure 7:**
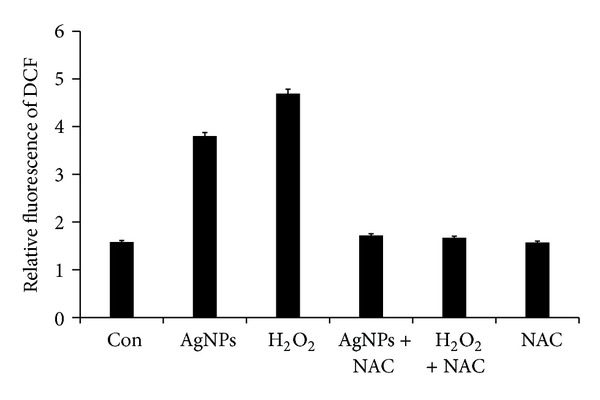
ROS generation in AgNPs treated MDA-MB-231 cells. Relative fluorescence of DCF was measured using a spectrofluorometer with excitation at 485 and emission at 530 nm. The results represent the means of three separate experiments, and error bars represent the standard error of the mean. Treated groups showed statistically significant differences from the control group by the Student's *t*-test (*P* < 0.05).

To know the effect of AgNPs in oxidative stress, we measured ROS generation using the H2DCF-DA assay. AgNPs induced intracellular ROS generation was evaluated using intracellular peroxide-dependent oxidation of DCFHDA to form fluorescent DCF. Cells were also treated with a characteristic ROS generating agent, H_2_O_2_ (1 mM), as a positive control. DCF fluorescence was detected in cells treated with AgNPs for 24 h. As shown in Figure 7, the ROS levels generated in response to AgNPs were significantly higher in AgNPs treated cells than control. ROS generation in cells treated with both AgNPs and H_2_O_2_ was decreased when the cells were pretreated with NAC, an antioxidant. Taken together, all these results indicate that cell death is mediated by ROS production, which might alter the cellular redox status, and it is a potential reason for cell death.

### 3.8. Caspase-3 Activation of AgNP-Induced Apoptosis

**Figure 8:**
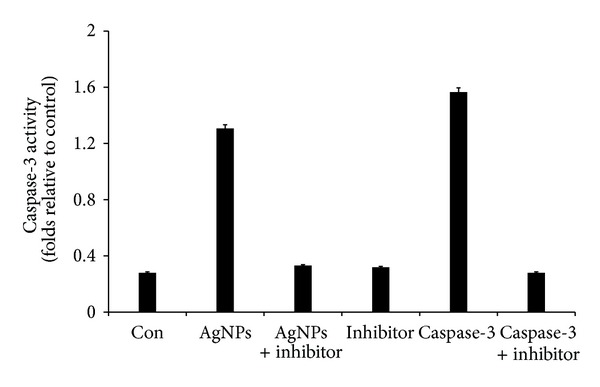
AgNPs induce apoptosis in MDA-MB-231 cells by caspase-3 activation. MDA-MB-231 cells were treated with AgNPs, purified caspase-3, and caspase-3 inhibitor for 24 h. The assay was performed as described in Materials and Methods Section. The caspase-3 activity is based on the hydrolysis of caspase-3 substrate (acetyl-Asp-Glu-Val-Asp p-nitroanilide (Ac-DEVD-pNA) by caspase-3, resulting in the release of the p-nitroaniline (pNA) moiety. The concentration of the pNA released from the substrate is calculated from the absorbance values at 405 nm. The assay was carried out in the presence of purified caspase-3 and caspase-3 inhibitor (Ac-DEVD-CHO) for a comparative analysis. The results represent the means of three separate experiments, and error bars represent the standard error of the mean. Treated groups showed statistically significant differences from the control group by the Student's *t*-test (*P* < 0.05).

The caspase-3 activation cascade plays an important role in several apoptotic mechanisms [65–67]. To investigate the potential effect of AgNP on apoptotic pathway, we examined the activity of caspase-3 in AgNP treated MDA-MB-231 cells.  Figure 8 depicts the increase in the levels of caspase-3 during treatment with AgNPs. The IC 50 value of AgNPs 8.7 *μ*g/mL increased the activity of caspase-3 to a level comparable with that of caspase-3 activation. The cellular metabolic activity seems to be affected by the AgNPs therefore, the possibility of apoptosis induction by the AgNPs was assessed, especially at the IC50. Levels of caspase-3, a molecule which plays a key role in the apoptotic pathway of cells, were increased following the treatment with AgNPs. The increased level of caspase 3 activation suggested that AgNPs caused cell death through apoptosis. 

### 3.9. DNA Fragmentation

**Figure 9:**
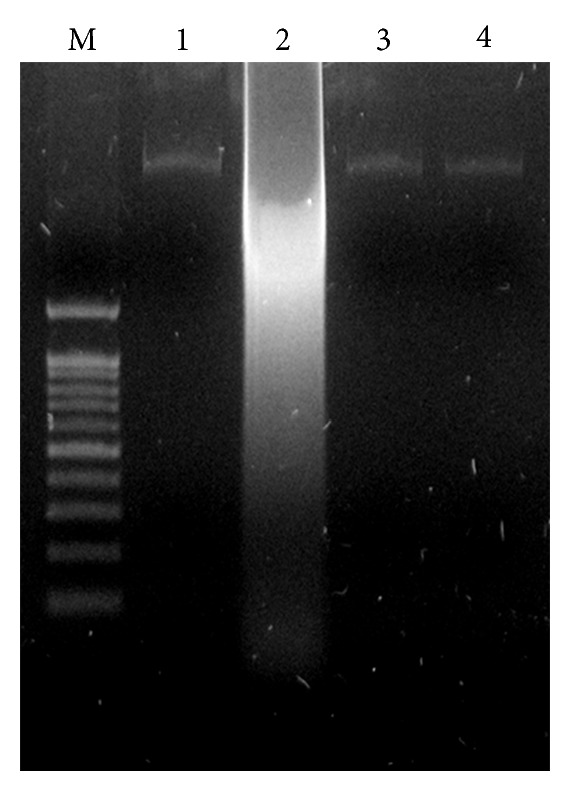
Effect of AgNPs on DNA fragmentation. MDA-MB-231 Cells were treated with AgNPs for 24 h and DNA fragmentation was analyzed by agarose gel electrophoresis. Lane M, 1 kB ladder; lane 1, control; lane 2, AgNP (8.7 *μ*g/mL); lane 3, AgNP + NAC; lane 4, NAC (5 mM).

The DNA laddering technique is used to visualize the endonuclease cleavage products of apoptosis [46]. This assay involves extraction of DNA from a lysed cell homogenate followed by agarose gel electrophoresis. Apoptosis of the AgNP treated cells was accompanied by a reduction in the percentage of cells in G0/G1 phase and an increase in the percentage of G2/M phase cells, indicating cell cycle arrest at G2/M [60]. The ROS can act as signal molecules promoting cell cycle progression and can induce oxidative DNA damage [68, 69]. Further we examined the impact of AgNPs in DNA fragmentation. DNA fragmentation is broadly considered as a characteristic feature of apoptosis [70]. Induction of apoptosis can be confirmed by two factors such as irregular reduction in size of cells, in which the cells are reduced and shrunken, and lastly DNA fragmentation. The DNA fragmentation in the present study was verified by extracting DNA from MDA-MB-231 cells treated with various concentrations of AgNPs followed by detection in the agarose gel.  Figure 9 clearly indicates that the DNA “laddering” pattern in MDA-MB-231 cells treated with AgNPs is one of the reasons for cell death. Earlier studies by Gurunathan and coworkers demonstrated that both cancer and noncancer cell lines treated with silver nanoparticle exhibit the formation of DNA ladder [14, 29]. The deposition of metal particles inside the nucleus could affect the DNA and cell division. Genotoxic studies of titanium dioxide (TiO_2_) nanoparticles revealed dose-dependent DNA damage, chromosomal aberrations and errors in chromosome segregation [71], and formation of sister chromatic exchanges [72]. Treatment with AgNPs induced the production of micronuclei (MN) [27]. Mroz et al. [73] hypothesized that nanoparticles and reactive oxidative species induce DNA damage, activating p53 and proteins related to DNA repair, and mimicking irradiation related carcinogenesis.

## 4. Conclusion

Recently AgNPs are used as an antimicrobial agent in wound dressings and coatings in medical devices. Developing biocompatible molecule as an anticancer agent is one of the novel approaches in the field of cancer therapy using nanotechnology. We have successfully synthesized and prepared stable AgNPs (20 nm) using novel bacterium, *B. funiculus*, which is green, environmentally friendly, cost effective, and rapid method for synthesis of AgNPs. The present study revealed that the potential cytotoxic effect of biologically synthesized AgNP in MDA-MB-231 cells by inhibiting growth of cells concentration-dependent activation of LDH increased level of ROS generation and activation of caspase-3, which is considered to be the most significant of the executioner caspases resulting in cellular apoptosis. Our results suggest that oxidative stress seems to be involved in nanoparticle cytotoxicity. The overall results indicated that the biologically synthesized AgNPs have antiproliferative activity through induction of apoptosis in MDA-MB-231 breast cancer cell line, suggesting that biologically synthesized AgNPs might be a potential alternative agent for human breast cancer therapy. This study demonstrates the possibility of using AgNPs to inhibit the growth of the tumor cells and their cytotoxicity for potential therapeutic treatments and offers a new method to develop molecule for cancer therapy. Finally, cost effectiveness, biocompatibility, and facileness to modify these silver nanoparticles make them a viable choice in future biomedical applications.
